# Dihydromyricetin ameliorates hepatic steatosis and insulin resistance via AMPK/PGC-1α and PPARα-mediated autophagy pathway

**DOI:** 10.1186/s12967-024-05060-7

**Published:** 2024-03-26

**Authors:** Yan Yang, Wen Qiu, Jiyuan Xiao, Jie Sun, Xuan Ren, Luxia Jiang

**Affiliations:** 1 Department of Endocrinology and Metabolism, Lanzhou University Second Hospital, Lanzhou, China; 2 Department of Pharmacology, Lanzhou University Second Hospital, Lanzhou, China; 3 Department of Cardiac Surgery ICU, Lanzhou University Second Hospital, Lanzhou, China

**Keywords:** Dihydromyricetin, Liver steatosis, Insulin resistance, Autophagy

## Abstract

**Background:**

Dihydromyricetin (DHM), a flavonoid compound of natural origin, has been identified in high concentrations in *ampelopsis grossedentata* and has a broad spectrum of biological and pharmacological functions, particularly in regulating glucose and lipid metabolism. The objective of this research was to examine how DHM affected nonalcoholic fatty liver disease (NAFLD) and its underlying mechanisms involved in the progression of NAFLD in a rat model subjected to a high-fat diet (HFD). Additionally, the study examines the underlying mechanisms in a cellular model of steatohepatitis using palmitic acid (PA)-treated HepG2 cells, with a focus on the potential correlation between autophagy and hepatic insulin resistance (IR) in the progress of NAFLD.

**Methods:**

SD rats were exposed to a HFD for a period of eight weeks, followed by a treatment with DHM (at doses of 50, 100, and 200 mg·kg^−1^·d^−1^) for additional six weeks. The HepG2 cells received a 0.5 mM PA treatment for 24 h, either alone or in conjunction with DHM (10 µM). The histopathological alterations were assessed by the use of Hematoxylin–eosin (H&E) staining. The quantification of glycogen content and lipid buildup in the liver was conducted by the use of PAS and Oil Red O staining techniques. Serum lipid and liver enzyme levels were also measured. Autophagic vesicle and autolysosome morphology was studied using electron microscopy. RT-qPCR and/or western blotting techniques were used to measure IR- and autophagy-related factors levels.

**Results:**

The administration of DHM demonstrated efficacy in ameliorating hepatic steatosis, as seen in both in vivo and in vitro experimental models. Moreover, DHM administration significantly increased GLUT2 expression, decreased G6Pase and PEPCK expression, and improved IR in the hepatic tissue of rats fed a HFD and in cells exhibiting steatosis. DHM treatment elevated Beclin 1, ATG 5, and LC3-II levels in hepatic steatosis models, correlating with autolysosome formation. The expression of AMPK levels and its downstream target PGC-1α, and PPARα were decreased in HFD-fed rats and PA-treated hepatocytes, which were reversed through DHM treatment. AMPK/ PGC-1α and PPARα knockdown reduced the impact of DHM on hepatic autophagy, IR and accumulation of hepatic lipid.

**Conclusions:**

Our findings revealed that AMPK/ PGC-1α, PPARα-dependent autophagy pathways in the pathophysiology of IR and hepatic steatosis has been shown, suggesting that DHM might potentially serve as a promising treatment option for addressing this disease.

**Graphical Abstract:**

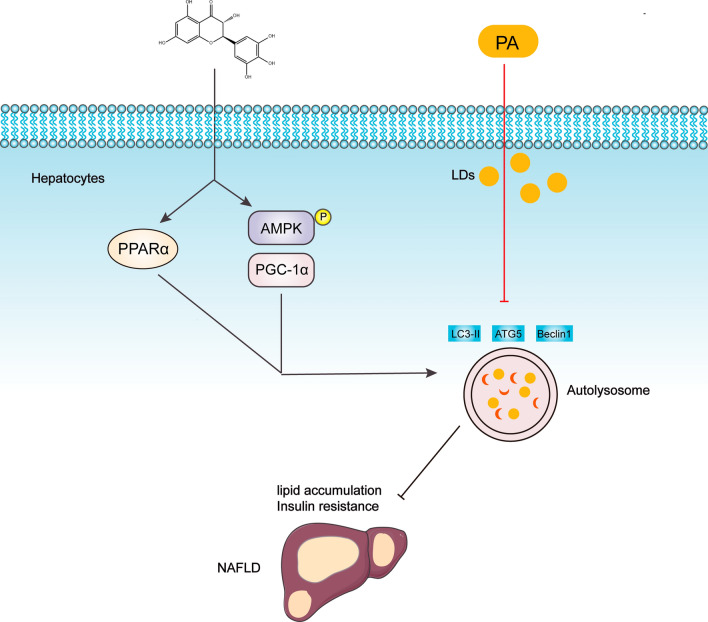

## Introduction

Nonalcoholic fatty liver disease (NAFLD) is a commonly seen chronic hepatic condition, with a prevalence rate of around 25% among the general population [1]. The incidence of NAFLD among the Asian population was 29.62% in 2019 [2]. The condition is distinguished by the excessive buildup of triglycerides (TG) inside hepatocytes. NAFLD is a condition characterized by clinical and pathological manifestations that encompasses two distinct conditions: non-alcoholic fatty liver and non-alcoholic steatohepatitis (NASH). If left untreated, NAFLD has the potential to progress to the development of liver cirrhosis and hepatocellular carcinoma [3]. NAFLD is a potential risk factor to increase susceptibilities to type 2 diabetes mellitus, cardiovascular disease, and chronic kidney disease [4]. However, up to now, there is a lack of authorized pharmaceutical interventions for NAFLD prevention and therapy, emphasizing the imperative nature of exploring the pathophysiology of NAFLD and develop potentially effective therapeutic targets [5].

Insulin resistance (IR) is the primary cause of NAFLD and may persist throughout the die. The release of an excessive amount of free fatty acids (FFA) occurs, leading to their entry into the circulation and subsequent uptake by the liver due to the failure of insulin-mediated suppression of lipolysis in adipose tissues during IR. Moreover, insulin promotes de novo synthesis of liver lipids, progressing to NAFLD [6]. Insulin sensitizers, such as metformin, improve insulin sensitivity by inhibiting hepatic glucose production and increasing the clearance of blood sugar. Therefore, metformin have been shown to be a promising drug for the treatment of NAFLD due to its metabolic benefits [7]. In addition to the benefits, metformin also has side effects including gastrointestinal disturbances, vitamin B_12_ deficiency, and lactic acidosis.

Numerous studies have demonstrated that various flavonoid phytochemicals significantly improve IR. Plant foods, such as fruits and vegetables, are rich sources of flavonoids [8]. Remarkably, flavonoids have minimal side effects compared to chemical drugs [9, 10]. Hence, these compounds have lately been recognized as prospective candidates for the prevention and treatment of NAFLD. Dihydromyricetin (DHM), sometimes referred to as ampelopsin, is the predominant flavonoid found in *Ampelopsis grossedentata*. This plant species is often used in southern China for the production of vine tea, with the delicate stems and leaves being the primary components. Notably, DHM accounts for around 20–30% of the vine tea's dry weight [11]. DHM exhibit multiple pharmacological activities, including antioxidant, antitumor, hypoglycemic, hypolipidemic, and hepatoprotective properties [12–14]. Therefore, IR improvement through DHM has a high potential application in preventing and treating NAFLD, as well as providing an experimental basis for developing natural hypoglycemic drugs.

Autophagy is a crucial cellular mechanism involved in maintaining homeostasis, wherein various components inside the cytoplasm, such as dysfunctional organelles and improperly folded proteins, are selectively delivered to lysosomes for destruction [15]. An increasing amount of research indicates that autophagy is significantly contributed to the control of homeostasis in lipid metabolism. Autophagosomes identify lipid droplets (LDs) and then facilitate their degradation through the process of lipophagy inside lysosomes [16]. The autophagy is shown to be compromised in the hepatic tissues of individuals diagnosed with NAFLD, as well as in mouse models of NAFLD [17]. Furthermore, it has been shown that hepatic autophagy is suppressed in mice with obesity-induced IR as a result of diminished expression of autophagy-related genes or the proteolytic cleavage of essential autophagy proteins by the calpain protease. Additionally, animals with targeted deletion of autophagy in particular tissues exhibit IR [16, 18, 19]. The results of these research suggest that the dysregulation of autophagy has a significant role in IR and the progression of NAFLD. Therefore, autophagy induction is considered a key treatment regimen in NAFLD.

The primary aim of this research was to evaluate the underlying protective mechanism of DHM compared to metformin against NAFLD in rats that were administered a high-fat diet (HFD), and in HepG2 cells were subjected to palmitic acid (PA) exposure. The results revealed that DHM is equally effective as metformin in alleviating hepatic IR and reducing hepatic steatosis by inducing autophagy.

## Materials and methods

### Animal experiments

The tests were conducted in adherence to the animal experimentation rules established by the National Institute of Animal Health. A total of forty-two specific pathogen-free (SPF) male Sprague Dawley (SD) rats, at an age of eight weeks, the subjects exhibited an average weight of 200 ± 20 g were housed together inside cages under controlled environmental conditions. These conditions included a temperature of 22 ± 2 ℃, a 12-h light/dark cycle, and unlimited accessibility to food and water. The rats were randomized to two groups, namely the normal control (NC) group with a sample size of 7 and the HFD group with a sample size of 35, using a random assignment method. The control group of rats received a regular rat normal chow meal, whereas the HFD group was provided with a HFD consisting of 60% kcal fat, specifically the D12492 diet from Research Diets. Following a period of eight weeks of feeding, samples of blood were acquired through the tail vein in order to determine fasting blood glucose (FBG) and insulin levels. Additionally, the homeostasis model assessment of insulin resistance [HOMA-IR = FBG (mmol/L) × fasting serum insulin (mIU/L)/22.5] was calculated. The effective modeling of IR in rats was deemed a significant differential between the group subjected to a HFD and the group serving as the control group. Then, one rat and three rats were randomly selected from NC and HFD group respectively, and their liver tissues were stained by hematoxylin–eosin (HE). In the HFD group, histomorphological changes such as hepatocyte bullous steatosis and inflammatory responses were observed in the liver tissues of the three rats, confirming the successful construction of the NAFLD model. Next, the HFD-fed rats were randomly allocated into five groups in accordance with the following diets and fed for a period of 6 weeks. The groups included: HFD, rats fed with a high fat diet; MET, rats fed with a high fat diet supplemented with metformin (150 mg/kg/day); DHM-L, rats fed with a high fat diet supplemented with low-dose DHM (50 mg/kg/day); DHM-M, rats fed with a high fat diet supplemented with medium-dose DHM (100 mg/kg/day); DHM-H, rats fed with a high fat diet supplemented with high-dose DHM (200 mg/kg/day). At the same time, the rats in the NC group were fed a normal chow diet for another 6 weeks. The DHM sample was prepared by dissolving it in a 0.5% solution of NaCMC, whereas the metformin sample served as a positive reference. The study spanned 14 weeks, during which the body weight and food consumption of each rat were evaluated weekly. Following the tests for insulin and glucose tolerance, all of the rats were euthanized after an overnight fast. For further analysis, samples of plasma and liver tissue were collected.

### Glucose tolerance and insulin tolerance tests

After a duration of six weeks of DHM medication, the intraperitoneal glucose tolerance test (IPGTT) and insulin tolerance test (IPITT) were conducted. For the IPGTT, rats underwent an overnight fast and were then intraperitoneally injected with 2 g of glucose per kg of body weight. For IPITT, The rats had a fasting period of 6.5 h, after which they were administered recombinant human insulin Novolin R (Novo Nordisk) via intraperitoneal injection at a concentration of 1 U/kg. Glucometer measurements were taken to assess tail vein blood glucose levels were measured at five time points: 0, 30, 60, 90, and 120 min during both IPGTT and IPITT. The area under glycemic curve (AUC) for each rat was calculated. The estimation of hepatic insulin sensitivity was conducted using the quantitative insulin-sensitivity check index (QUICKI), which was derived as 1/log (fasting insulin [mU/L]) + log (fasting glucose [mg/dL]) [20, 21].

### Serum biochemical analysis

Total cholesterol (TC), aspartate aminotransferase (AST), alanine aminotransferase (ALT), high-density lipoprotein cholesterol (HDL-C), and low-density lipoprotein cholesterol (LDL-C) concentrations were measured using the VITROS® 5600 automatic biochemistry analyzer (Johnson and Johnson). The serum FFA and insulin levels and TG concentration in hepatocytes were assessed by using enzyme-linked immunosorbent assay (ELISA) kits in strict adherence to the manufacturer's directions.

### Histopathological analysis

The liver tissues were subjected to a 24-h fixation period in 4% polyformaldehyde. Subsequently, the tissues were processed for paraffin embedding and sectioned into 4 μm-thick slices. Histopathological examination was performed after dewaxing and staining with HE. The sections were subjected to Periodic Acid-Schiff (PAS) staining, which included the use of periodic acid solution and Schiff's reagents (G1008, Google, Wuhan, China) to detect glycogen content. Additionally, the liver sections, frozen with 8 μm thickness, were exposed to incubation with a freshly diluted solution of Oil Red O staining for a duration of 10 min. Subsequently, the sections were washed in a solution containing 60% isopropanol and PBS in order to detect the accumulation of LDs. The HepG2 cells were subjected to fixation using a 4% paraformaldehyde solution for 15 min. Following that, the cells underwent staining using Oil Red O for 10 min. Following the staining process, the cells were rinsed with a solution consisting of 60% isopropanol and PBS. Finally, the cells were observed under a microscope.

### Electron microscopy

The tissue slices, measuring 1 mm^3^, were treated with fixation by immersion in a buffered glutaraldehyde solution with a concentration of 2.5%. Subsequently, they were dehydrated using a series of ethanol concentrations and ultimately embedded in Epon 812 (CAS number 90529–77-4, SPI, USA). The observation of autophagic vacuoles and autolysosomes was conducted using a transmission electron microscope manufactured by Hitachi, Japan.

### Cell culture and treatments

The Dulbecco's modified Eagle's medium (DMEM), supplemented with 10% fetal bovine serum (FBS) and 1% penicillin–streptomycin solution, was used to cultivate the HepG2 cell line, which was derived from human hepatoma cells. Both DMEM and FBS solutions were sourced from HyClone, USA. The cells were maintained at a temperature of 37 ℃ in a 5% CO2 incubator. The growth media were supplemented with PA ( P9767, Sigma-Aldrich) at a concentration of 0.5 mM to create an in vitro lipid-loaded cell model mimicking NAFLD. In the context of in vitro tests, the cells were subjected to an overnight incubation in a serum-free medium, followed by a 24-h treatment with or without DHM (5,10, and 20 µM) in media containing PA or vehicle.

### SiRNA-mediated knockdown

Six-well tissue culture plates with a seeding density of 2.5 × 10^3^ HepG2 cells per were used for the culture of the cells. The cells were allowed to grow until the cells achieved a confluence of 80% on a growth medium without antibiotics, but supplemented with FBS. SiRNA for PGC-1α and PPARα was purchased from Genentech, China, while siRNA_AMPK_ was designed as previously described [22] (Table 1). Lipofectamine 2000, produced by Thermo Scientific Inc. located in Virginia, was used to transfect specific siRNA into the cells. The transfection procedure was carried out successfully following the manufacturer's instructions. The cells in the control group were subjected to transfection with scrambled siRNA. The efficacy of gene silencing was assessed by quantifying the quantities of mRNA and protein expression.

**Table 1:** Oligonucleotide sequences of siRNAs

siRNA	Sequences:sense(5'-3')	Sequences:complement strand(5'-3')
AMPK-siRNA	AGUGAAGGUUGGCAAACAU	AUGUUUGCCAACCUUCACU
PPARα-siRNA1	GCGUAUGGAAAUGGGUUUAUAdTdT	UAUAAACCCAUUUCCAUACGCdTdT
PPARα-siRNA2	GCGAUCUAGAGAGCCCGUUAUdTdT	AUAACGGGCUCUCUAGAUCGCdTdT
PPARα-siRNA3	GCAGAAAUUCUUACCUGUGAAdTdT	UUCACAGGUAAGAAUUUCUGCdTdT
PGC1α-siRNA1	GACUAUUGCCAGUCAAUUAAUdTdT	AUUAAUUGACUGGCAAUAGUCdTdT
PGC1α-siRNA2	CGACUUGGAUACAGACAGCUUdTdT	AAGCUGUCUGUAUCCAAGUCGdTdT
PGC1α-siRNA3	GACAGCGAAGAUGAAAGUGAUdTdT	AUCACUUUCAUCUUCGCUGUCdTdT

### Real-time quantitative reverse-transcription polymerase chain reaction (qRT-PCR)

The process of extracting total RNA from liver tissue and HepG2 cells was conducted using TRIzon (Invitrogen, Carlsbad, CA, USA). Utilizing 1 µg of total RNA, complementary DNA (cDNA) was produced, using the reverse transcription apparatus provided by TransGen (China) according to the manufacturer's procedure. The PCR amplification was performed using a pre-mixed Taq reaction mixture including SYBR Green dye, in addition to gene-specific primers as described in Table 2. The obtained data were subjected to normalization using the reference gene GAPDH. The data underwent analysis using the 2^−∆∆CT^ methodology.

**Table 2:** Primers for quantitative RT-PCR analysis

Species	Gene	Primer sequence (5'-3')
Rattus norvegicus	G6Pase	Forward: TCAACCTCGTCTTCAAGTGGATT
		Reverse: CTGCTTTATTATAGGCACGGAGCT
Rattus norvegicus	PEPCK	Forward: GGCGGAGCATATGCTGATCC
		Reverse: CCACAGGCACTAGGGAAGGC
Rattus norvegicus	GLUT2	Forward: CTCGGGCCTTACGTGTTCTTCCTT
		Reverse: TGGTTCCCTTCTGGTCTGTTC
Rattus norvegicus	PPARα	Forward: TCGCAGGAAAGACTAGCAAC
		Reverse: ATGCACAAGGTCTCCATGTC
Rattus norvegicus	PGC-1α	Forward: ATGAGAAGCGGGAGTCTGAA
		Reverse: ACGGTGCATTAATCAATTTC
Rattus norvegicus	BECN1	Forward: AGTTGCCGTTGTACTGTTCTG
		Reverse: TCAATCTTGCCTTTCTCCAC
Rattus norvegicus	ATG5	Forward: TGAACGAGAAGCAGAGCCATAC
		Reverse: TTGGATAATGCCATTTCAGG
Rattus norvegicus	LC3	Forward: CATCAACATTCTGACGGAGCG
		Reverse: GTTGCTTGGCATCAAACACG
Rattus norvegicus	GAPDH	Forward: TCGTGGAGTCTACTGGCGTCTT
		Reverse: CATTGCTGACAATCTTGAGGGAG
Homo sapiens	G6Pase	Forward: GTACAGGGAGAGCTGCAAGG
		Reverse: AGGACGAGGGAGGCTACAAT
Homo sapiens	PEPCK	Forward: CTGCCCAAGATCTTCCATGT
		Reverse: CAGCACCCTGGAGTTCTCTC
Homo sapiens	GLUT2	Forward: GGCTGAGGAAGAGACTGTGG
		Reverse: ACAGACAGGGACCAGAGCAT
Homo sapiens	AMPK	Forward: CTTTGGCAGTTGCCTACCAT
		Reverse: GGCTTGTCGCCAAATAGAAA
Homo sapiens	PPARα	Forward: CAATGCACTGGAACTGGATG
		Reverse: GCAAATGATAGCAGCCACAA
Homo sapiens	PGC-1α	Forward: CCTTGCAGCACAAGAAAACA
		Reverse: TGACCGAAGTGCTTGTTCAG
Homo sapiens	BECN1	Forward: GCTGGCACTAGAGGAGGAGA
		Reverse: CGGTTCTTTTCCACGTCTTC
Homo sapiens	ATG5	Forward: AAAGATGTGCTTCGAGATGTGT
		Reverse: CACTTTGTCAGTTACCAACGTCA
Homo sapiens	LC3	Forward: CCTTCTTCCTGCTGGTGAAC
		Reverse: CTCGTCTTTCTCCTGCTCGT
Homo sapiens	GAPDH	Forward: GGAGCGAGATCCCTCCAAAAT
		Reverse: GGCTGTTGTCATACTTCTCATGG

### Western blot analysis

The process of extracting total protein from liver tissue and hepatocytes was carried out at a temperature of 4℃ using RIPA lysis buffer (P0013B, Beyotime, China) that was supplemented with 0.1% protease inhibitor cocktail (P8340, Sigma-Aldrich, St. Louis, MO, USA). 30 µg of protein samples were separated through SDS-PAGE (Bio-Rad Laboratories, USA) and transferred onto PVDF membranes (Millipore, Billerica, MA, USA). The membranes were blocked using a 5% solution of skimmed milk and then underwent overnight incubation at a temperature of 4 °C with primary antibodies, diluted at a ratio of 1:1,000. After the washing procedure with TBST, The membranes were subjected to incubation with secondary antibodies coupled with horseradish peroxidase at a dilution ratio of 1:5,000 at room temperature for a duration of 1 h. The detection of membrane signals was accomplished by the use of an improved chemiluminescence technique, specifically using the Minichemi 610 instrument manufactured in China. Band intensities were quantified using ImageJ program. Table 3 enumerates the principal antibodies.

**Table 3:** Primary antibodies for western blot analysis

Name	Supplier	Cat no.
G6Pase	Bioss, China	bs-13253R
PEPCK	Proteintech, USA	14892-1-AP
GLUT2	Proteintech, USA	20436-1-AP
AMPK	Proteintech, USA	10929-2-AP
p-AMPK	Cell Signaling Technology, USA	2531S
PPARa	Proteintech, USA	15540-1-AP
PGC-1α	Proteintech, USA	66369-1-Ig
BECN1	Proteintech, USA	11306-1-AP
LC3	Proteintech, USA	14600-1-AP
ATG5	Proteintech, USA	10181-2-AP

### Statistical analysis

All experiments were independently conducted for three times. The data obtained from the experiments were analyzed using the SPSS program (version 27.0) developed by IBM Corp. Initial analysis involved performing a one-way analysis of variance (ANOVA) to assess differences between the groups. To further evaluate these differences between every two groups, Tukey post-hoc multiple comparisons were employed (*P* < 0.05). Statistical figures were then generated using GraphPad software (version 9.5.1). The quantitative data was presented in the form of mean ± standard deviation (SD).

## Results

### DHM attenuated HFD-induced NAFLD rats

**Fig. 1:**
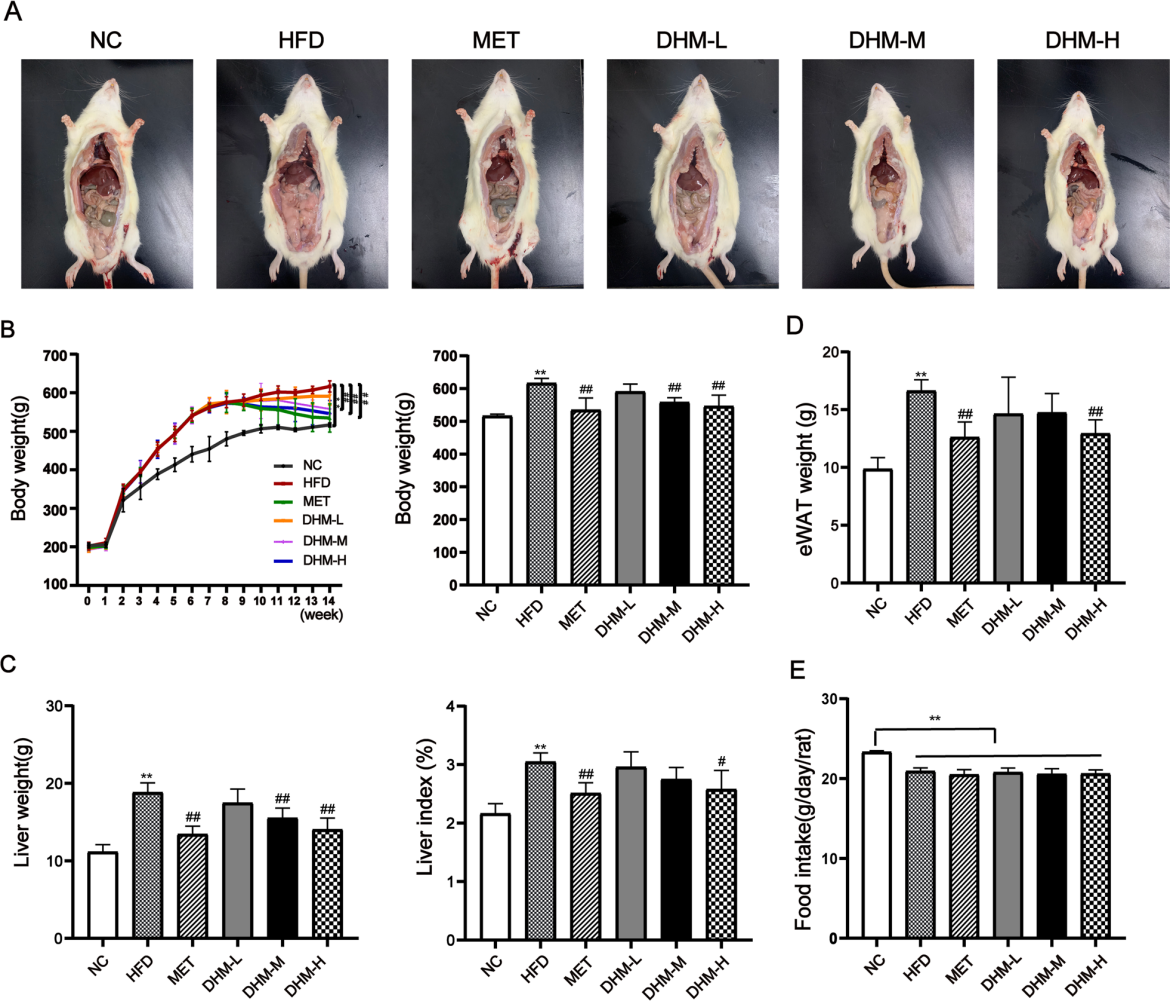
DHM attenuated HFD-induced NAFLD in rats. **A** Representative macroscopic images of rats. **B** Weight changes over 14 weeks and final body weight. Several NAFLD-related parameters were measured at week 14 in different groups. The parameters included **C** liver weight and liver index, **D** eWAT weight, and **E** food intake. Data are presented as mean ± SD (n = 6). ^*^P < 0.05, ^**^P < 0.01 *vs*. the NC group; ^#^*P* < 0.05, ^##^*P* < 0.01 *vs*. the HFD group

SD rats were fed a HFD or a standard chow diet for a duration of eight weeks. Subsequently, the rats underwent an additional six-week intervention with three different dosages of DHM (50 mg, 100 mg, and 200 mg/kg/day) or metformin (150 mg/kg/day) to explore the potential beneficial effects of DHM on NAFLD. Body weight and food consumption in rats were recorded weekly. Liver tissue and epididymal white adipose tissue (eWAT) were harvested and weighed after sacrifice. After 14 weeks of HFD, the HFD group exhibited statistically significant increases in body weight, liver weight, liver index, and eWAT weight compared to the NC group. Moreover, the body size of rats in HFD group was obviously larger than that in NC group. In contrast, DHM administration, especially DHM-H, effectively reversed the effects of HFD feeding (Fig. 1A–D), and did not show significant change compared to metformin group (*P* > 0.05). It was observed that rats on an HFD had reduced appetite due to the high-fat content during the rat rearing process. Therefore, the HFD group had a significantly lower average food intake than the control group. However, there was no statistically significant difference in food intake between the HFD and the DHM groups (Fig. 1E).

### The administration of DHM demonstrated a reduction in hepatic steatosis and damage in rats with NAFLD and palmitic acid-treated HepG2 cells

**Fig. 2:**
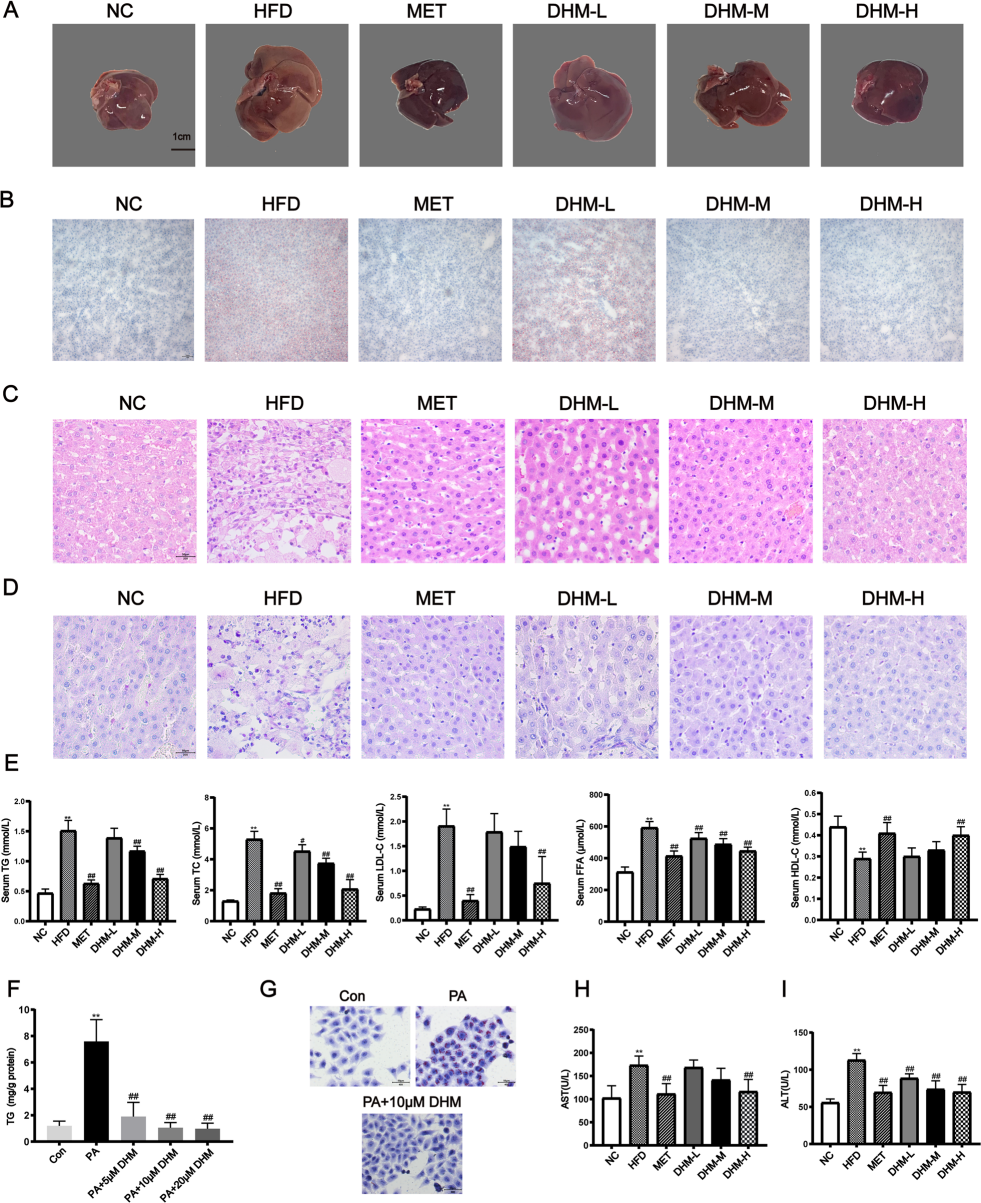
DHM attenuates hepatic steatosis and injury in NAFLD rats and palmitic acid-treated HepG2 cells. **A** Representative macroscopic pictures of the rat liver from each experimental group are shown. **B**–**D** Representative liver slices were subjected to staining with Oil Red O, as well as with H&E and PAS. Scale bar = 50 μm. **E** Serum lipid concentration (n = 6). ^*^ *P* < 0.05, ^**^ *P* < 0.01 *vs*. the NC group; ^#^ *P* < 0.05, ^##^ *P* < 0.01 *vs*. the HFD group. The HepG2 cell line was subjected to treatment with PA (0.5 mM) and/or DHM at concentrations of 5, 10, or 20 μM. for 24 h. **F** Measurement of intracellular TG concentration. **G** Oil Red O staining under a light microscope. Scale bar = 50 μm. Data are presented as mean ± SD of triplicate experiments. ^**^P < 0.01 *vs*. untreated control. ^##^*P* < 0.01 *vs*. PA-treated cells. (H, I) Serum ALT and AST levels (n = 6). ^*^P < 0.05, ^**^P < 0.01 *vs.* the NC group; ^#^*P* < 0.05, ^##^*P* < 0.01 *vs.* the HFD group

Liver morphological and histological examinations (Oil red O staining and H&E) were conducted to investigate the existence of hepatic steatosis, a defining feature of NAFLD to confirm the effects of DHM. The rat liver's macroscopic pictures demonstrated that the intake of a HFD led to a significant increase in liver size and a shift in liver color from a dark hue to a paler shade (Fig. 2A). The Oil red O staining and H&E results indicated that the hepatic tissue of rats that were administered a HFD for a duration of 14 weeks exhibited pronounced hepatic steatosis, ballooning degeneration, inflammatory infiltration, and necrosis (Fig. 2B, C). Compared to the HFD group, the high-dose DHM group had less hepatocyte swelling, lipid droplet accumulation, inflammatory cell infiltration, and no ballooning degeneration, with a reddish-brown surface and a soft texture, exhibiting the same effect as the MET group. Although the DHM-L group exhibited the existence of LDs and inflammatory infiltration, the overall state of the hepatocytes was comparatively superior than that of the HFD group.

Liver glycogen storage was assessed by staining cells with PAS. There was a significant reduction in glycogen content observed in the model group compared to the control group, as seen in Fig. 2D. After a six-week treatment period, it was observed that the groups administered with middle-dose and high-dose of DHM had a significant increase in glycogen content compared to the model group. Moreover, the high-dose DHM group exhibited similar effects as the MET group (positive control).

Given that lipid metabolism disturbance is a prominent etiological factor in NAFLD, our research focused on evaluating the potential protective effects of DHM against this condition by analyzing conventional biochemical markers. Following a 14-week intervention including a HFD, the blood levels of total TC, TG, LDL-C, and FFA exhibited a statistically significant increase in the HFD group. Conversely, the level of HDL-C was a statistically significant reduction when compared to the NC group. DHM supplementation significantly improved the serum lipid profile abnormalities induced by a HFD feeding dose-dependently (Fig. 2E). In the high-dose DHM group, compared to the HFD group, the levels of TC, TG, and LDL-C were reduced by 60.8%, 53%, and 60.7% respectively. Based on the observed raised blood lipid levels and histological results in rats fed with a HFD, it was shown that the TG content and LDs in the HepG2 cells exhibited a statistically significant increase in the group that received treatment with PA in comparison to the control group. However, DHM administration significantly reduced these PA-induced increases, indicating that DHM could ameliorate hepatocellular lipid accumulation and steatosis (Fig. 2F, G). Both 10 and 20 uM concentrations of DHM significantly reduced TG content compared to the PA treatment group, with no significant difference between the two dosages (Fig. 2F). HepG2 cells were treated with 10 uM of DHM for the subsequent experiments.

In order to examine the potential of DHM in mitigating liver cell damage induced by a HFD, the levels of serum transaminases were assessed. These transaminases are well recognized as biomarkers for evaluating liver injury. At the conclusion of the 14th week, the blood levels of ALT and AST in the HFD group had a statistically significant rise in comparison to the NC group. DHM treatment reduced HFD-induced plasma ALT and AST elevation in a dose-dependent manner. The results of this investigation provide empirical support for the hepatoprotective properties of DHM in the context of HFD consumption (Fig. 2H, I).

### DHM improved glucose homeostasis and insulin sensitivity

**Fig. 3:**
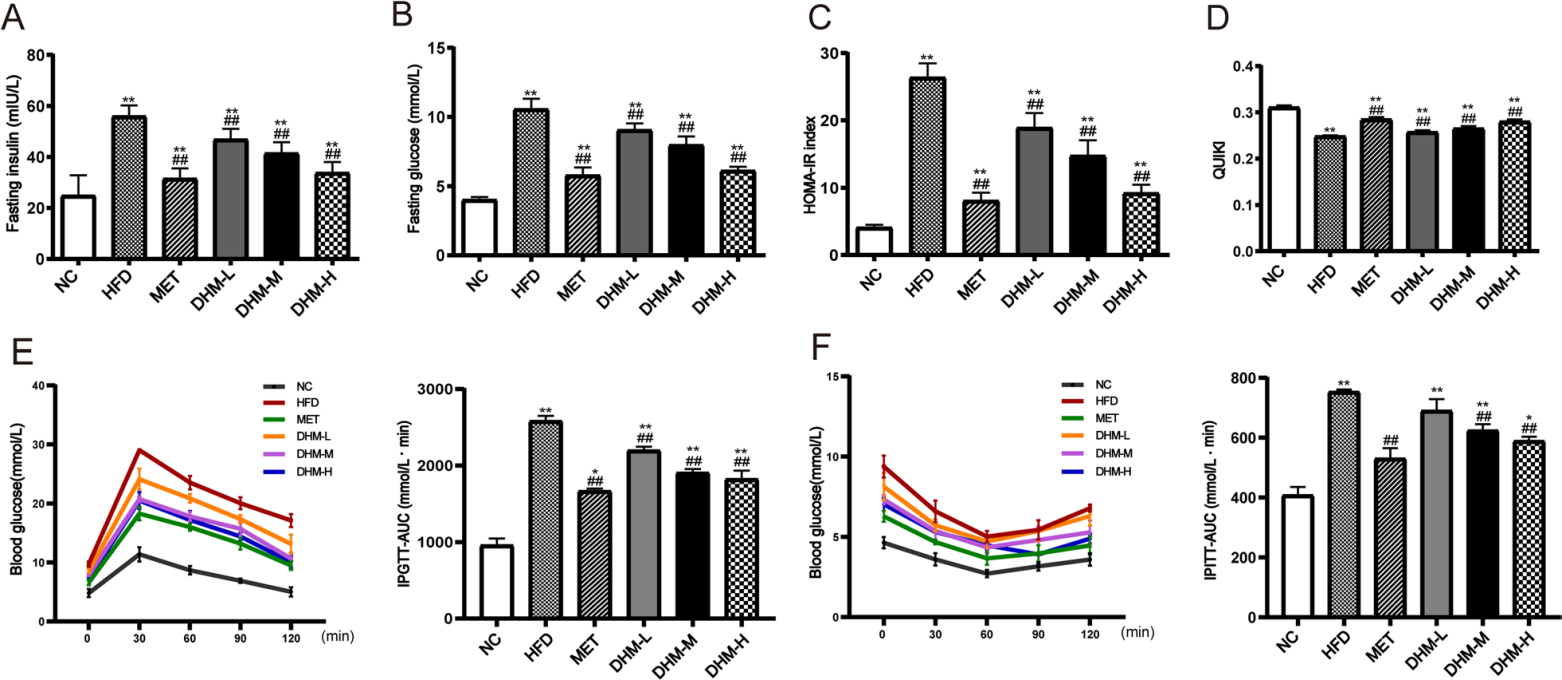
The administration of DHM resulted in enhanced regulation of glucose levels inside the body and increased responsiveness to insulin. At week 14, measurements were taken for several parameters correlated to glucose homeostasis in the six groups of rats (n = 6). The parameters included: **A** fasting blood insulin; **B** fasting blood glucose; **C** homeostasis model assessment (HOMA)-insulin resistance index; **D** The quantitative insulin-sensitivity check index (QUICKI); **E** The blood glucose curve and the corresponding area under the curve in the IPGTT (n = 3); **F** The blood glucose curve and area under the curve in the IPITT (n = 3). ^*^P < 0.05, ^**^P < 0.01 *vs*. the NC group; ^#^*P* < 0.05, ^##^*P* < 0.01 *vs*. the HFD group

Glucose homeostasis and IR are crucial in NAFLD assessment. After 14 weeks of HFD feeding, the levels of fasting blood insulin (Fig. 3A), FBG (Fig. 3B), and the HOMA-IR (Fig. 3C) indicated that the HFD group had a greater increase in comparison to the NC group, while demonstrating a decrease in the DHM or MET group. Moreover, the QUICKI level was significantly suppressed by HFD; however, this effect was prevented by DHM or MET supplementation (Fig. 3D). Moreover, the AUC values for both the IPGTT and IPITT showed that the HFD group exhibited higher levels of the aforementioned factors in comparison to the NC group. Furthermore, the AUC values were consistently decreased by treatment with DHM or metformin (Fig. 3E, F). We observed that, although the biomarkers remained different from the normal control group following the intervention with DHM or MET, there was a significant improvement in comparison to the HFD group. The findings of the study indicated that DHM can improve glucose tolerance and insulin sensitivity in rats that were given a HFD.

### DHM regulated the expression of hepatic insulin resistance-related factors in HFD-induced rats and palmitic acid-treated HepG2 cells

**Fig. 4:**
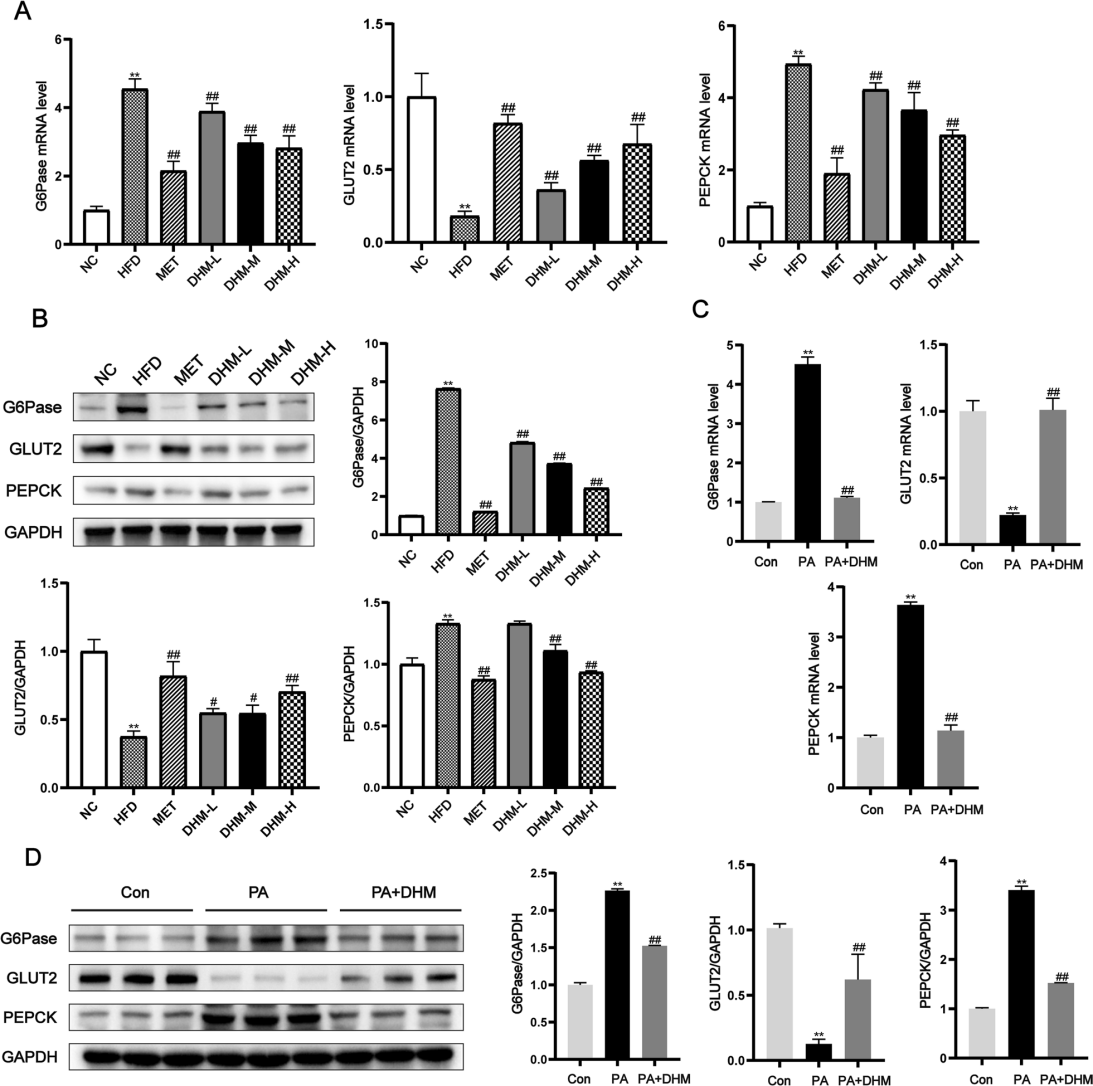
DHM regulated the expression of hepatic insulin resistance-related factors in HFD-induced rats and palmitic acid-treated HepG2 cells. **A** The mRNA levels and **B** the protein expressions of G6Pase, GLUT2, and PEPCK were assessed by the use of RT-qPCR and western blot analysis in the livers of six groups of rats fed normal chow diet (NC), HFD, HFD with DHM (DHM-L, DHM-M, DHM-H), and HFD with meiformin (MET), respectively (n = 3). ^*^P < 0.05, ^**^P < 0.01 *vs*. the NC group; ^#^*P* < 0.05, ^##^*P* < 0.01 *vs*. the HFD group. For 24 h, HepG2 cells were treated with PA (0.5 mM) and DHM (10 uM). **C** G6Pase, GLUT2, and PEPCK mRNA levels were determined by RT-qPCR. **D** Western blot analysis of G6Pase, GLUT2, and PEPCK protein expression. ^*^P < 0.05, ^**^P < 0.01 *vs*. the Con group; ^#^*P* < 0.05, ^##^*P* < 0.01 *vs*. the PA group. The protein quantities were determined by comparing them to the quantity of GAPDH protein

The key enzymes regulating liver gluconeogenesis are glucose-6-phosphatase (G6Pase) and phosphoenolpyruvate carboxykinase (PEPCK). The level of glucose transporter type 2 (GLUT2), a major indicator of insulin signaling pathway activity, plays a crucial function in facilitating glucose uptake upon insulin activation in the liver and inhibiting glucose release from hepatocytes. The mRNA and protein expression levels of G6Pase, PEPCK, and GLUT2 were quantified using RT-qPCR and western blot techniques to assess the influence of DHM on hepatic IR (refer to Fig. 4A, B). The hepatic expression of PEPCK and G6Pase mRNA and protein was significantly higher in the group of rats given an HFD compared to those on an NC diet. Conversely, the hepatic expression of GLUT2 was significantly decreased in rats fed a HFD. However, DHM dose-dependently inhibited the expression of G6Pase and PEPCK while increasing the expression of GLUT2. The administration of PA therapy resulted in a statistically significant upregulation of G6Pase and PEPCK mRNA and protein expression levels, while concurrently inducing a downregulation of GLUT2 mRNA and protein expression levels in HepG2 cells, correlating with animal experiments. Notably, DHM treatment reversed these FFA-induced effects significantly (Fig. 4C, D). These results suggest that DHM may improve hepatic IR by suppressing hepatic glucose production.

### DHM induced autophagy in HFD-induced rats and palmitic acid-treated HepG2 cells

**Fig. 5:**
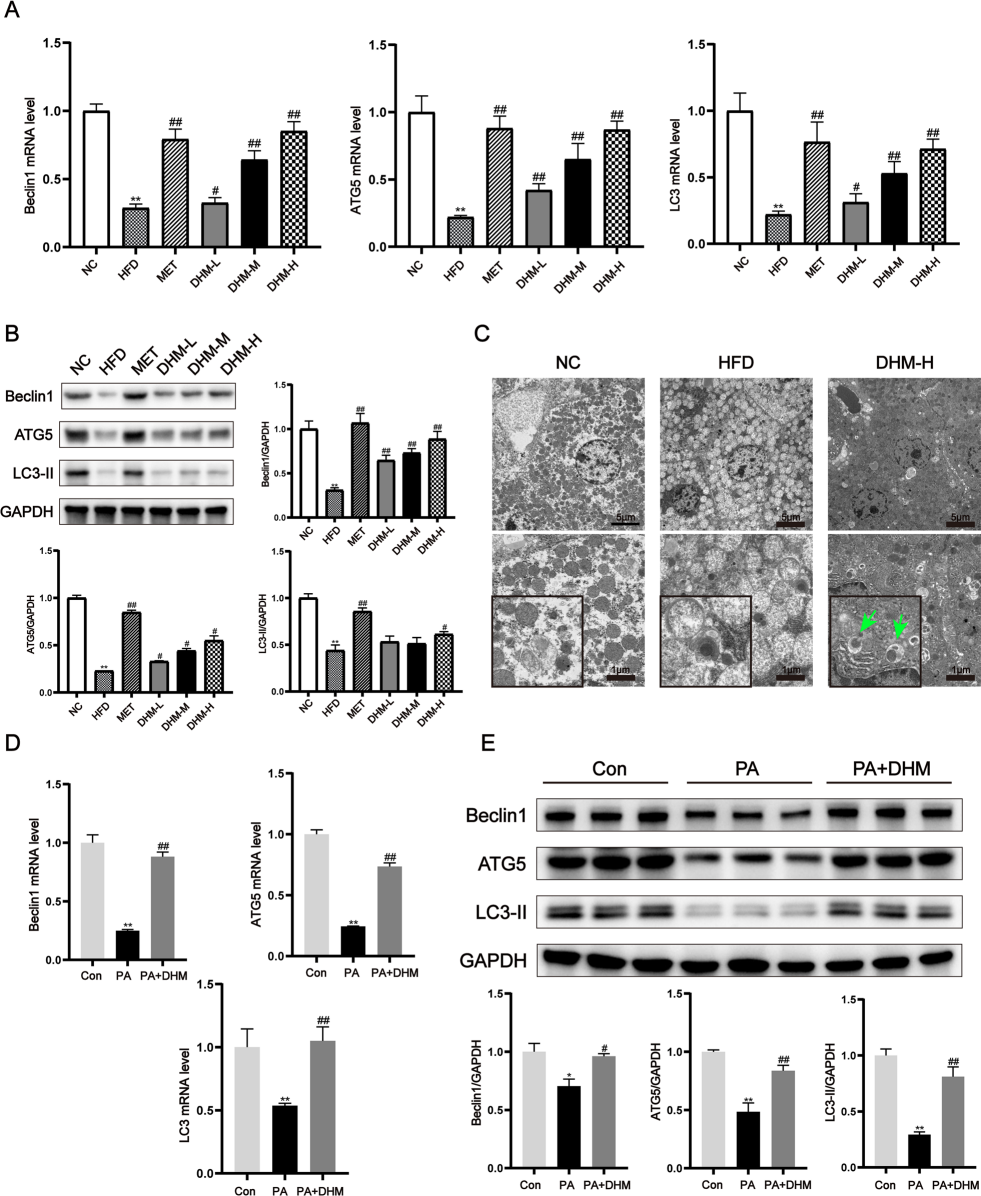
DHM induced autophagy in HFD-induced rats and palmitic acid-treated HepG2 cells. **A** The mRNA levels and **B** The protein expressions of autophagy indicators were assessed by the use of both RT-qPCR and western blot techniques. This analysis was conducted on the livers of six distinct groups of rats that were provided with a NC, HFD, HFD with DHM (DHM-L, DHM-M, DHM-H), and HFD with metformin (MET), respectively (n = 3). ^*^P < 0.05, ^**^P < 0.01 *vs*. the NC group; ^#^*P* < 0.05, ^##^*P* < 0.01 *vs*. the HFD group. **C** Images of autophagic vacuoles in the liver obtained using electron microscopy. Autolysosomes are shown by arrows. For 24 h, HepG2 cells were treated with PA (0.5 mM) and DHM (10 uM). **D** The mRNA levels and protein expressions of autophagy markers were assessed using RT-qPCR and western blot techniques, respectively. ^*^P < 0.05, ^**^P < 0.01 *vs*. the Con group; ^#^*P* < 0.05, ^##^*P* < 0.01 *vs*. the PA group

Previous research has shown autophagy has a vital function in the pathogenesis of hepatic steatosis and is suppressed in the hepatic tissues of persons with NASH and animals subjected to a HFD, similar to HFD-induced rats in our study. Interestingly, the mRNA and protein levels of myosin-like BCL2-interacting protein (Beclin) 1, autophagy-related gene (ATG) 5 and microtubule-associated protein light chain 3 (LC3)-II, well-established markers of autophagy induction, increased in a dose-dependent manner after DHM administration in HFD rats (Fig. 5A, B). Moreover, transmission electron microscopy revealed more autophagic vacuoles in the liver of DHM (DHM-H)-treated rats compared to the HFD group (Fig. 5C). Similaly, the treatment of PA resulted in a decrease in Beclin1, ATG5, and LC3-II mRNA and protein expression in HepG2 cells. Nevertheless, the administration of DHM resulted in the reversal of these effects (Fig. 5D, E). The findings of this study show that DHM had a preventive effect on hepatic steatosis via the activation of autophagy.

### AMPK, PGC-1α, and PPARα activation contributed to DHM's hepatoprotective action against lipotoxicity

**Fig. 6:**
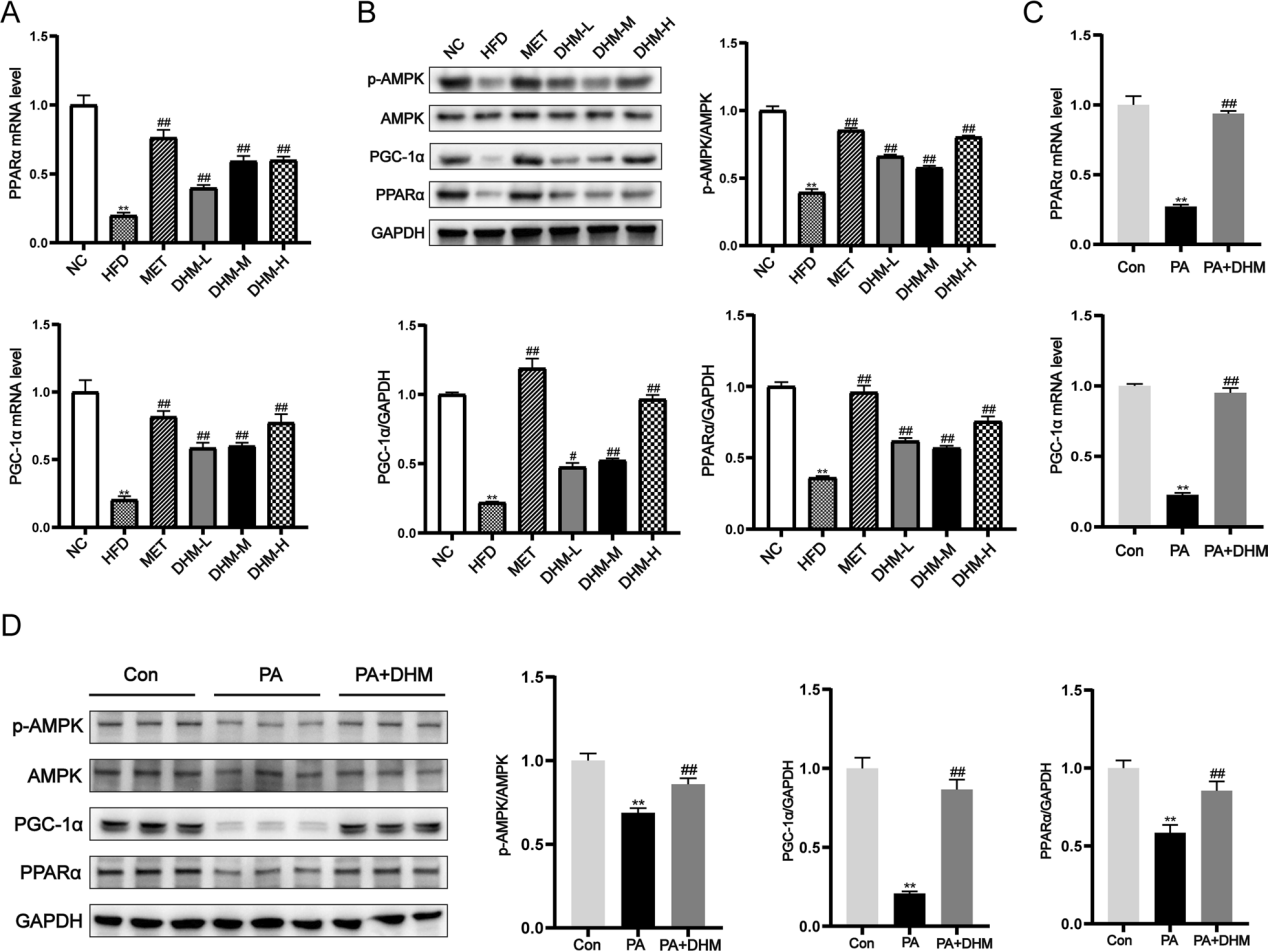
AMPK, PGC-1α, and PPARα activation contributed to DHM hepatoprotective action against lipotoxicity. **A** The mRNA levels of PGC-1α and PPARα, and **B** the protein expressions of AMPK、PGC-1α and PPARα were analyzed using RT-qPCR and western blot in livers of six groups of rats fed normal chow diet (NC), HFD, HFD with DHM (DHM-L, DHM-M, DHM-H), and HFD with metformin (MET), respectively (n = 3). ^*^P < 0.05, ^**^P < 0.01 *vs*. the NC group; ^#^*P* < 0.05, ^##^*P* < 0.01 *vs*. the HFD group. For 24 h, HepG2 cells were treated with PA (0.5 mM) and DHM (10 μM). (**C**) The mRNA levels of PGC-1α and PPARα and (**D**) protein expressions of AMPK, PGC-1α and PPARα were analyzed using RT-qPCR and western blot, respectively. ^*^P < 0.05, ^**^P < 0.01 *vs*. the Con group; ^#^*P* < 0.05, ^##^*P* < 0.01 *vs*. the PA group

Peroxisome proliferator-activated receptor γ coactivator-1α (PGC-1α), AMP-activated protein kinase (AMPK), and peroxisome proliferator-activated receptor α (PPARα) activation play roles in suppressing hepatic steatosis through multiple pathways [23–26]. To determine whether AMPK, PGC-1α, and PPARα are targets of DHM, we investigated the impacts of DHM on hepatic p-AMPK、PGC-1α and PPARα abundance in HFD-fed rats and PA-exposed HepG2 cells. HFD-feeding resulted in a decrease in the hepatic PPARα and PGC-1α mRNA expression, and the protein expression of p-AMPK, PGC-1α, and PPARα exhibited significant changes (Fig. 6A, B). DHM administration rescued HFD-induced reduction of hepatic p-AMPK, PGC-1α, and PPARα in a dose-dependent manner. PA downregulated the expression of PPARα and PGC-1α mRNA, and p-AMPK, PGC-1α and PPARα proteins in HepG2 cells. However, DHM-treated HepG2 cells demonstrated a significant improvement in PA-induced p-AMPK, PGC-1α and PPARα downregulation (Fig. 6C, D). These results demonstrate that p-AMPK, PGC-1α, or PPARα upregulation, is required for DHM to ameliorate liver lipotoxic injury.

### PGC-1α was the downstream target of DHM-triggered AMPK activation

**Fig. 7:**
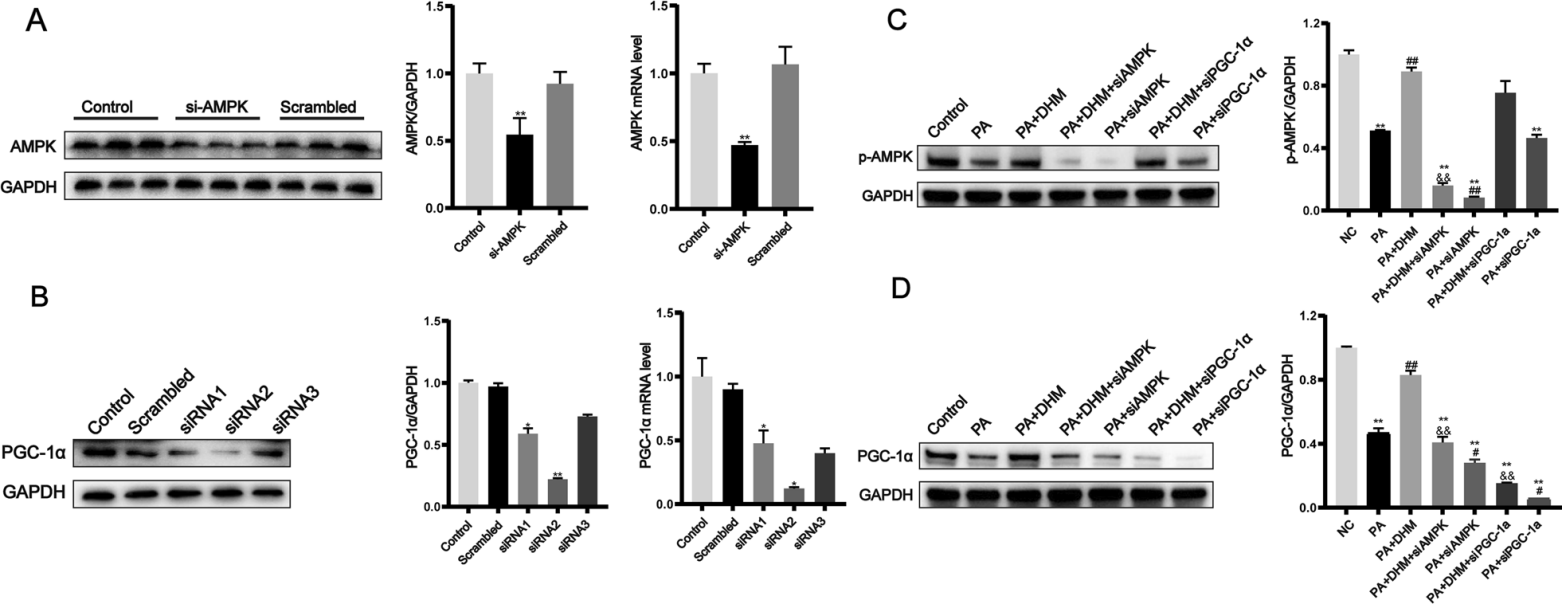
The downstream target of DHM-triggered AMPK activation was PGC-1α. For 24 h, HepG2 cells were transfected with AMPK-specific or scrambled siRNA. RT-qPCR or western blot were used to evaluate AMPK mRNA and protein expression. **B** HepG2 cells were transfected for 24 h with PGC-1α-specific siRNA (three pairs of sequences, designated siRNA1, siRNA2 and siRNA3, respectively) or scrambled siRNA. The mRNA and protein levels of PGC-1α were measured using RT-qPCR or western blot. Interference sequences with the highest silencing efficiency were selected to silence PGC-1α gene expression in subsequent experiments. Cells were treated with 0.5 mM PA for 24 h with or without DHM (10 μM) pretreatment after siRNA silencing of AMPK or PGC-1α. Western blot was performed for (**C**) p-AMPK or (**D**) PGC-1α. ^*^P < 0.05, ^**^P < 0.01 *vs*. the Con group; ^#^*P* < 0.05, ^##^*P* < 0.01 *vs*. the PA group; ^&^*P* < 0.05, ^&&^*P* < 0.01 *vs*. the PA + DHM group

The regulatory relationship between AMPK and PGC-1α was analyzed using gene silencing. AMPK and PGC-1α gene silencing through siRNA transfection was performed; the effectiveness of siRNA knockdown is seen in Fig. 7A, B. The results showed that AMPK silencing suppressed DHM-activated PGC-1α upregulation significantly, whereas PGC-1α gene silencing through siRNA transfection had no effect on DHM-induced p-AMPK activation (Fig. 7C, D), suggesting that PGC-1α was a downstream target of DHM-induced AMPK activation.

### DHM induced autophagy via AMPK/PGC-1α and PPARα pathway

**Fig. 8:**
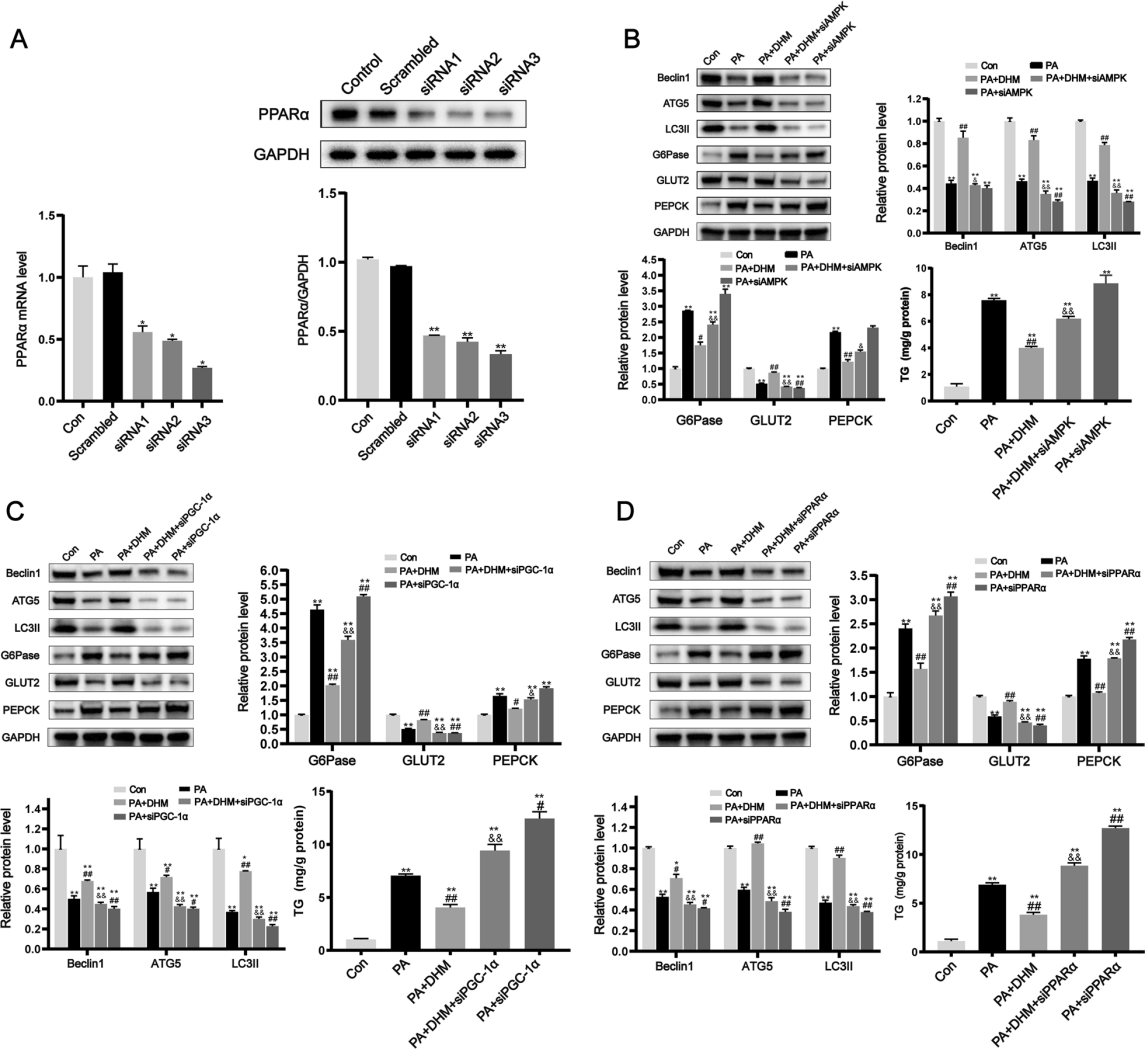
DHM induced autophagy via the AMPK/PGC-1α and PPARα pathways. **A** HepG2 cells were transfected with PPARα-specific siRNA (three pairs of sequences, designated siRNA1, siRNA2, and siRNA3) or scrambled siRNA for 24 h. RT-qPCR and western blot were used to determine PPARα mRNA and protein expression. Interference sequences with the highest silencing efficiency were selected to silence PPARα gene expression in subsequent experiments. After siRNA silencing of AMPK (**B**), PGC-1α (**C**), or PPARα, (**D**) cells were treated with 0.5 mM PA for 24 h with or without pretreatment with DHM (10 μM). Western blot was used to analyze the protein expressions of autophagy markers, insulin resistance-related factors, and intracellular TG content. *P < 0.05, **P < 0.01 vs. the Con group; ^#^P < 0.05, ^##^P < 0.01 vs. the PA group; ^&^P < 0.05, ^&&^P < 0.01 vs. the PA + DHM group

Previous studies have demonstrated that AMPK, PGC-1α, or PPARα induce autophagy and mitigate lipid accumulation in both in vivo and in vitro [23, 27–30]. Therefore, we investigated whether DHM treatment activated autophagy by modulating the AMPK/PGC-1α and PPARα pathway in liver. PPARα gene silencing was conducted by means of siRNA transfection, as seen in Fig. 8A, which illustrates the efficacy of siRNA knockdown. The administration of DHM effectively restored the reduced levels of Beclin1, ATG5, and LC3-II proteins caused by PA (Fig. 8B–D); however, AMPK, PGC-1α, or PPARα silencing through respective siRNA transfection abolished DHM-induced autophagic activity, as evidenced by reduced Beclin1、ATG5, and LC3-II expression levels. The assessment of intracellular TG levels showed that DHM effectively mitigated the TG buildup generated by PA. The increased TG content in hepatocytes indicated that AMPK, PGC-1α, or PPARα gene silencing blocked the ameliorative effect of DHM on lipid accumulation in the liver. Moreover, AMPK, PGC-1α, or PPARα silencing abrogated the beneficial effects of DHM on the upregulation of G6Pase and PEPCK expression, and the downregulation of GLUT2 expression in HepG2 cells treated with PA. These results indicate that AMPK/PGC-1α, PPARα-related signaling pathways are important in mediating DHM-induced autophagy in NAFLD rats. Moreover, the role of DHM in reducing hepatic IR and fat accumulation is partially attributed to autophagy-inducing AMPK/PGC-1α, PPARα-related pathways.

## Discussion

NAFLD is a prevalent chronic condition that is characterized by IR and the excessive buildup of lipids in hepatocytes. This is subsequently accompanied by oxidative stress, damage to mitochondria, infiltration of inflammatory cells, and disruption of the gut microbiota. These factors all contribute to a "multi-hit" effect on the liver [31]. In this study, we discovered that DHM, a traditional Chinese Medicine (TCM), effectively reduced body weight, fat mass, liver weight, and liver index, significantly improving pathological lesions in liver tissues caused by HFD. Moreover, DHM treatment reduced the elevated serum levels of AST, ALT, FFA, TG, LDL-C, and TC in NAFLD rats. DHM also effectively inhibited the accumulation of hepatic lipid in HFD-fed rats and steatotic hepatocytes. Its effect is comparable to that of insulin sensitizer metformin. Therefore, DHM might be a potential treatment option for NAFLD in experimental animal models.

There was no significant reduction in food consumption noted during the six weeks of DHM treatment in our study. Therefore, we hypothesized that the beneficial effect of DHM on weight loss was not dependent on the inhibition of food intake, especially for short-term interventions. Hence, it is plausible to trace the systemic metabolic effects of DHM to other processes.

As observed in another study [32], the HFD-induced rat NAFLD models developed severe IR. Our results revealed that the HOMA-IR and AUC values obtained from both the IPGTT and IPITT procedures were seen to exhibit a statistically significant increase in the HFD group compared to the NC group, while hepatic insulin sensitivity (QUICKI) was impaired, which was consistently ameliorated by DHM. Hepatic IR is defined by a reduction in the efficacy of insulin in its capacity to inhibit the production of glucose inside the liver and decreased hepatic glycogen synthesis, increasing gluconeogenesis and hepatic glucose output. G6Pase and PEPCK are the key liver enzymes regulating the conversion of non-sugar substances into glucose during gluconeogenesis. Their increased expression is associated with enhanced gluconeogenesis. GLUT2, the most abundant GLUT isoform in hepatocytes, is responsible for most of the glucose uptake. The expression and function of GLUT2 on the liver membrane are affected by a high-fat environment, and glucose utilization in the liver is insufficient, resulting in hepatic IR. In the current study, we discovered that DHM ameliorated hepatic gluconeogenesis and stimulated glucose uptake, as indicated by the downregulation of G6pase and PEPCK expression and the upregulation of GLUT2 expression. DHM also increased glycogen content in hepatocytes. These results indicated that DHM played an important role in reducing hepatic IR in HFD-fed rats.

The underlying processes responsible for the protective impact of DHM on hepatic steatosis and IR were investigated. Autophagy is a highly conserved recycling process responsible for maintaining cellular metabolism and energy homeostasis. The impairment of autophagy has been identified as a putative determinant in the pathogenesis of NAFLD, since it may contribute to the buildup of fat in the liver, as well as damage, inflammation, fibrosis, and the development of liver cancer. These processes play significant role in the pathogenesis of NAFLD [33, 34]. Increasing evidence indicates that TCM, as a novel autophagy enhancer, may improve hepatic steatosis and insulin sensitivity in HFD-induced obesity [35]. In the current study, transmission electron microscopy revealed an abundance of LDs in the liver of HFD rats. Besides, swelling and rupture of the mitochondrial ridge are observed, indicating significant mitochondrial damage. Furthermore, after DHM treatment, lipid-laden autolysosomes were observed instead of LDs, and mitochondrial damage was also alleviated. The findings of our investigation indicate that DHM administration resulted in a dose-dependent increase in the expression of autophagy markers, specially Beclin1, ATG5, and LC3-II, in both a rat model of NAFLD and a cellular model of hepatocytic steatosis. Thus, it suggested that DHM effectively reversed the inhibitory impacts of a high-fat diet on autophagy, potentially through mechanisms involving the autophagy-lysosomal pathway.

We then investigated how DHM affects the autophagy process. AMPK is an enzyme that belongs to the serine/threonine kinase family and is known for its pivotal involvement in the regulation of energy balance and the detection of nutritional levels inside the body. The activation of AMPK occurs via the process of phosphorylation at the Thr172 residue located in the α subunit [36]. The activation of AMPK hinders energy-consuming activities that rely on ATP, such as the synthesis of fatty acids and the production of glucose from non-carbohydrate sources. Conversely, it promotes energy-generating processes that contribute to ATP production, such as fatty acid oxidation and the breakdown of glucose via glycolysis. This control is achieved by the modulation of a transcriptional regulatory factor known as PGC-1α [36, 37]. Several bioactive compounds isolated from medicinal herbs attenuate the activation of AMPK that leads to the buildup of lipids in the liver [38]. PPARα plays a crucial role in the regulation of lipid metabolism in the liver [39]. In the human population, there exists a negative correlation between hepatic PPARα levels and NASH. Furthermore, an elevation in PPARα expression levels has been seen to be linked with histological improvement [40]. In a similar vein, PPARα^−/−^ mice had elevated levels of hepatic triglycerides, oxidative stress, inflammation, and cellular apoptosis, along with a substantially increased NAFLD activity score in the group given a HFD as compared to the wild-type controls fed HFD [41]. In addition, previous study revealed that vine tea polyphenols, DHM as one of its components, may protect mice from western diet containing high fat, sugar, and cholesterol induced NAFLD via moderating expression of PPARα [42]. Therefore, the expression of AMPK, PGC-1α, and PPARα were detected.

As previously observed [35, 43], p-AMPK, PGC-1α, and PPARα were downregulated in HFD-induced liver injury and FFA-induced hepatocyte steatosis. However, the group treated with DHM showed an upregulation of p-AMPK, PGC-1α, and PPARα levels compared to the untreated HFD group. AMPK phosphorylates PGC-1α directly both in vitro and in vivo [43]. We confirmed that PGC-1α is a downstream target of DHM-triggered AMPK activation, indicating that DHM might regulate AMPK/PGC-1α and PPARα signaling.

The hepatic lipid buildup is effectively inhibited by AMPK via the activation of autophagy, a catabolic mechanism responsible for the removal of defective macromolecules and organelles by lysosomal destruction [23]. The PPARα activation leads to the upregulation of many autophagy-related genes by its direct binding to their respective promoters [44]. We observed that silencing p-AMPK, PGC-1α, or PPARα prevented DHM-induced liver autophagy activation. These results suggest that AMPK/PGC-1α and PPARα-dependent autophagy might be the principal mechanism through which DHM regulates energy metabolism. Moreover, AMPK/PGC-1α or PPARα silencing through exclusive siRNA abrogated the effects of DHM on hepatic lipid accumulation and IR. These findings indicate that DHM might regulate NAFLD through multiple signaling pathways involving AMPK/PGC-1α and PPARα-induced autophagy.

However, we exclusively studied the major organ (liver) of rats with NAFLD and did not evaluate the modulatory effect of DHM on autophagy in other insulin-acting tissues, such as adipose tissue and skeletal muscle. Consequently, further research is necessary to explore the broader effects of DHM and other mechanisms that may be implicated.

## Conclusions

**Fig. 9:**
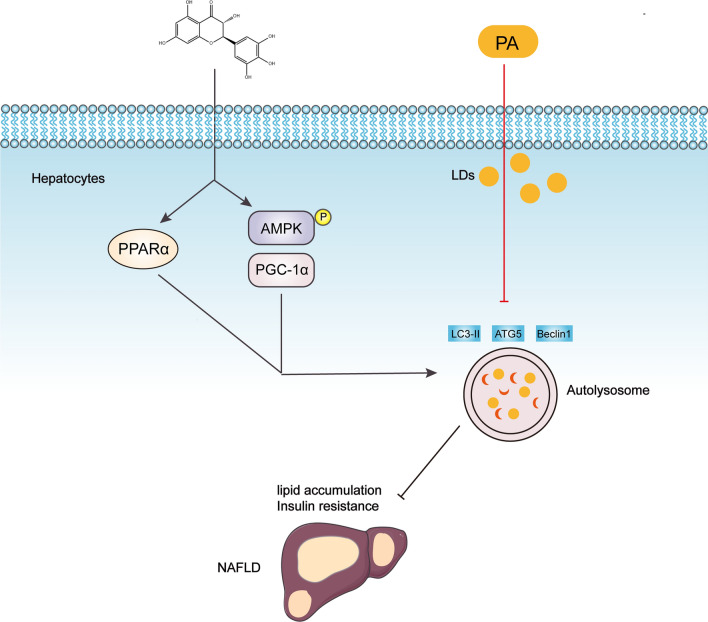
Schematic diagram of the proposed mechanisms for Dihydromyricetin ameliorating hepatic steatosis and IR in HFD-induced rats via the AMPK/PGC-1α and PPARα-mediated autophagy pathways

Although the underlying mechanisms of DHM in the prevention and treatment of NAFLD are complex, our findings indicate that DHM improves hepatic steatosis and IR via AMPK/PGC-1α or PPARα-inducing autophagy in a rat model and steatotic hepatocytes (Fig. 9). Currently, there are no FDA-approved pharmacological therapies for NAFLD. Therefore, DHM emerges as a promising therapeutic option for NAFLD in animals and could serve as a valuable reference and guide for clinical treatment.

## Data Availability

All data has been presented in this manuscript. All three tables are in the included Supplementary Material.

## Abbreviations

NAFLD: Non-alcoholic fatty liver disease
NASH: Non-alcoholic steatohepatitis
IR: Insulin resistance
FFA: Free fatty acids
DHM: Dihydromyricetin
IPGTT: Intraperitoneal glucose tolerance test
IPITT: Intraperitoneal insulin tolerance test
AST: Aspartate amino transferase
ALT: Alanine amino transferase
TG: Triglyceride
TC: Total cholesterol
LDL-C: Low density lipoprotein-cholesterol
HDL-C: High density lipoprotein-cholesterol
PA: Palmitic acid
eWAT: Epididymal white adipose tissue
FBG: Fasting blood glucose
G6Pase: Glucose-6-phosphatase
PEPCK: Hosphoenolpyruvate carboxykinase
GLUT2: Glucose transporter type 2
LDs: Lipid droplets
Beclin1: Myosin-like BCL2-interacting protein 1
ATG 5: Autophagy-related gene 5
LC3: Microtubule-associated protein light chain 3
AMPK: AMP-activated protein kinase
PGC-1α: Peroxisome proliferator-activated receptor γ coactivator-1α
PPARα: Peroxisome proliferator-activated receptor α
TCM: Traditional Chinese Medicine
